# Fatigue-Induced Damage in High-Strength Concrete Microstructure

**DOI:** 10.3390/ma14195650

**Published:** 2021-09-28

**Authors:** Nadja Oneschkow, Tim Scheiden, Markus Hüpgen, Corinna Rozanski, Michael Haist

**Affiliations:** Institute of Building Materials Science, Leibniz University Hannover, Appelstraße 9a, 30167 Hannover, Germany; t.scheiden@baustoff.uni-hannover.de (T.S.); m.huepgen@baustoff.uni-hannover.de (M.H.); c.rozanski@baustoff.uni-hannover.de (C.R.); haist@baustoff.uni-hannover.de (M.H.)

**Keywords:** compressive cyclic loading, high-strength concrete, strain development, acoustic emission, damage mechanism, light microscopy, SEM

## Abstract

A high-strength concrete subjected to compressive fatigue loading with two maximum stress levels was investigated and the behaviour was evaluated using the macroscopic damage indicators, strain and acoustic emission hits (AE-hits), combined with microstructural analyses utilising light microscopy and scanning electron microscopy (SEM). A clustering technique using Gaussian mixture modelling combined with a posterior probability of 0.80 was firstly applied to the AE-hits caused by compressive fatigue loading, leading to two clusters depending on the maximum stress level. Only a few cracks were visible in the microstructure using light microscopy and SEM, even in phase III of the strain development, which is shortly before failure. However, bluish impregnated areas in the mortar matrix of higher porosity or defects, changing due to the fatigue loading, were analysed. Indications were found that the fatigue damage process is continuously ongoing on a micro- or sub-microscale throughout the mortar matrix, which is difficult to observe on a mesoscale by imaging. Furthermore, the results indicate that two different damage mechanisms take place, which are pronounced depending on the maximum stress level. This might be due to diffuse and widespread compressive damage and localised tensile damage, as the findings documented in the literature suggest.

## 1. Introduction

The development and application of concretes with ever higher compressive strengths enable the realisation of more slender concrete structures. Compared to massive structures, these structures are exposed to fatigue-relevant loads to a higher extent because of their lower ratio of deadweight to non-static loads. Additionally, special structures, such as wind energy turbines or machine foundations, are generally exposed to huge numbers of load cycles. Thus, concrete fatigue behaviour has become an important field of research in the last few decades. The latest research, especially, is more focused on concrete fatigue behaviour or, rather, damage development, (see e.g., [1,2,3,4]). However, only a little knowledge is currently available concerning the characteristics of fatigue damage processes in the concrete microstructure.

The well-known s-shaped, three-phase development of the peak strain in force controlled fatigue tests is characteristic of the fatigue behaviour of concrete. The first phase is characterised by a disproportionate increase of strain, followed by a linear strain increase in phase II and a, once again, disproportionate strain increase up to failure in phase III. The transitions of phases depend on the type of cyclic fatigue loading and the kind of concrete and are roughly located in the area of 5–20%, in respectively 80–95% of the numbers of cycles to failure N_f_ [1,2,3,5,6,7]. Theories of ongoing processes in the microstructure have been derived based mainly on investigations of normal strength concretes. The first phase is associated with a disproportionate growth of microcracks, whereby an indication of plastic settlement in the first few load cycles was found (e.g., [3,8]). A stable growth of microcracks with a diffuse character is assumed for the second phase. In the third phase, an instable growth of cracks occurs, accompanied by the localisation and connection of microcracks forming macrocracks [3,8]. In [3], an indication was found that viscous deformations in the mortar matrix also take place, whereas Shah and Chandra assumed that the failure of concrete due to cyclic loading is caused by “…progressive internal cracking rather than by any viscous flow of the [hardened cement] paste” ([9], p. 819). However, cracks in the microstructure are difficult to find in the different phases of the compressive fatigue process (see e.g., [3,9]).

Continuously measurable, macroscopic damage indicators, such as concrete strain, stiffness or dissipated energy, are often used to describe the fatigue behaviour of concrete [2,3,4,5,10,11,12]. In addition, the acoustic emission is another continuously measurable damage indicator with high potential, enabling additional and strain-independent information on the damage process in the concrete microstructure to be obtained. Parameters, such as the amplitude, frequency and duration of acoustic emission hits (AE-hits), are often considered in terms of their quantitative distribution to provide additional information about damage propagation due to fatigue loading [3,13,14,15].

**Figure 1:**
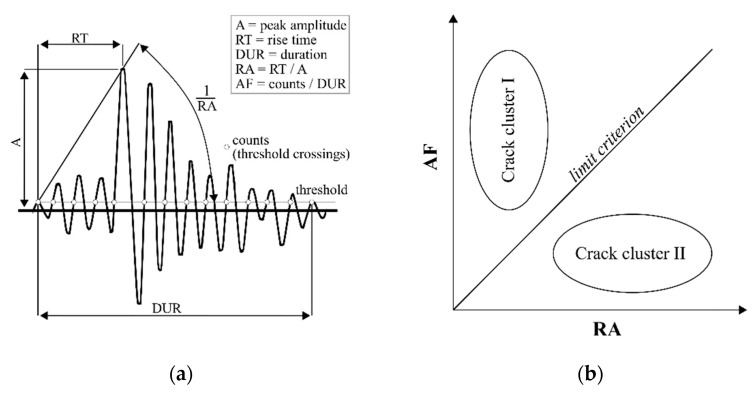
AE signal parameters (**a**); schematic diagram of clusters (**b**).

Aggelis [16] as well as Ohno and Ohtsu [17] related the AE-hits to two types of crack modes (tensile and shear) for reinforced concrete beams subjected to monotonically increasing bending loading with respect to the specification given in [18]. The objective of this (and other) investigations was to enable the monitoring of the remaining bearing capacity of existing reinforced concrete structures via acoustic emission. The characteristics of AE-hits are described by the acoustic emission parameters AF (average frequency) and RA as following (cf. also Figure 1a):

AF = (counts)/(duration)(1)

RA = (rise time)/(peak amplitude)(2)

Based on both parameters, following [18], the AE-hits can be divided into two clusters by means of a limit criterion (cf. Figure 1b). The limit criterion between AF and RA is not fixed and instead must be determined for each individual application. In its first applications for reinforced concrete beams the limit was drawn by observing the time when macroscopic failure occurred, e.g., [16,17,19]. Thus, AE-hits detected before the appearance of macroscopic failure were assigned to crack cluster I and AE-hits that arose at failure and afterwards were assigned to cluster II.

In Farhidzadeh et al. [20], the Gaussian mixture modelling in combination with a maximum likelihood estimation was applied for a better assignment of AE-hits to the clusters.

However, to the author’s knowledge, the clustering of AE-hits has only been applied for reinforced concrete elements with respect to meso- to macrocracks, which are visible to the naked eye. Thus, for compressive fatigue loading, which induces diffuse damage on a smaller scale, the general applicability of these clustering methods needs to be investigated more deeply. Furthermore, the transferability of the allocation of clusters to crack modes, as conducted for reinforced concrete beams (e.g., [17,19,20,21]), is questionable and, therefore, also needs an elementarily investigation.

However, investigation of the damage processes due to fatigue loading is a challenge because of the extremely small scale on which compressive fatigue damage occurs. Thus, visualisation of damage requires the utilisation of high-resolution imaging techniques, which are only applicable for discrete points in the damage process of many thousands of load cycles and, moreover, can only display extremely small regions of interest (ROIs) of the concrete microstructure. Thus, only locally and temporally discrete results are achievable. Furthermore, the tests have to be stopped and the specimens cut in order to prepare the samples for the investigations using high-resolution techniques. Load-induced cracks can generally hardly be clearly distinguished from those caused by hydration and shrinkage or by the sample preparation process. Micro X-ray computer tomography allows for a nondestructive investigation and could enable the tracking of cracks. However, the resolutions of commercially available instruments are often not sufficient due to the extremely small scale of compressive fatigue damage combined with the high-density of concrete and the necessary specimen diameters for fatigue tests on concrete and, therefore, often only enable the detection of relatively wide cracks shortly before failure (see e.g., [22,23,24]).

Regarding the degradation of the concrete microstructure under compressive fatigue loading, only limited information can be found in the literature due to the general limitations of the investigational methods described above. Cracks were found in the mortar matrix and the interfacial transition zone (ITZ) of normal strength concretes (see e.g., [9,25,26]), whereby the crack widths were not reported. A stress-level dependency of crack growth was found in the form of slower crack propagation for lower maximum stress levels [9] and fewer but wider cracks in phase III for higher maximum stress levels [25]. Shah and Chandra [9] assumed two stages of crack growth, which depend on the maximum stress level. Stage I crack growth is characterised by the absence of volume dilatation and occurs for concrete specimens, which did not fail, and for specimens of hardened cement paste. Stage II crack growth is characterised by accompanied volume dilatation and occurs at the end of the fatigue process of concrete specimens.

Ibuk [27] and Breitenbücher et al. [28] investigated polished sections of three normal strength concretes and Schäfer et al. [29] one high-strength concrete previously loaded under compressive fatigue. They found that mainly new cracks occurred rather than existing cracks being extended. The crack widths detected in [28,29] were in the range of 5 µm to 16 µm for the normal strength concretes and 5.9 µm to 6.4 µm for the high-strength concrete. Shah and Chandra [9] suggest that two different effects appear under compressive fatigue loading: consolidation with a consequent strengthening and cracking with a consequent weakening, whereas at higher loads, cracking may predominate over the strengthening effect [9].

Thiele [3] also investigated the crack formation in a normal strength concrete subjected to compressive fatigue loading using fluorescence microscopy with polished sections and scanning electron microscopy (SEM) with thin sections. Changes in the crack pattern could not be reliably detected in phase I and phase II. In phase III, the cracks were more orientated in the loading direction. However, indications were found that the crack length might decrease in the first phase, remain unchanged or increase slightly in phase II, and increase strongly in phase III. The mean crack widths increased slightly from phase I to phase II and strongly in phase III, with mean values in the range of 5.0 µm to 12.5 µm.

Thiele assumes that damage also occurs on a smaller scale than investigated (presumably smaller than 1 µm) because crack formation and propagation could not be reliably detected in phases I and II, but was detectable, e.g., by the development of stiffness and ultrasonic signals [3]. Previously, Oneschkow [1] had hypothesised that extremely small-scale structural changes in the concrete microstructure accumulate continuously and load dependently within the fatigue process as the pre-stages of damage visible on a mesoscale and constitute a boundary condition for the development of cracks and, therefore, affect the occurrence of transition to phase III, or rather, fatigue failure. However, this hypothesis was set up only based on the detailed analyses of strain and stiffness developments of a high-strength concrete due to different compressive fatigue loadings [1,2]. By using transmitted electron microscopy (TEM) together with a focused ion beam (FIB) sample preparation, Schaan et al. found needle- or lath-shaped regions of loosening or lower density in the mortar matrix of an ultra-high-strength concrete, which appeared after compressive fatigue loading on the nanoscale and rose in number within the fatigue process [30]. These findings support the hypotheses of both Oneschkow and Thiele.

Mehmel and Kern [31] and Lusche [32] developed models describing the character of stress distribution in the microstructure of normal strength concrete subjected to monotonically increasing [31,32] and cyclic compressive loads [32]. They understood the stiffer coarse aggregates as a disruption of the mortar matrix. This heterogeneity leads to an inhomogeneous stress distribution with areas of peak stresses in the mortar matrix, higher than the external stresses imposed, located next to the coarse aggregates. The magnitude of internal peak stresses depends on, for example, the ratio of the stiffness of the aggregate and mortar matrix, and the size and amount of aggregate. The comparison of the fatigue behaviour of cement stone and concrete in [31] showed that the fatigue behaviour of concrete is influenced significantly by the interaction of coarse aggregates and cement stone, a result which was confirmed by Shah and Chandra [9]. Mehmel and Kern [31] assumed that these areas of internal peak stresses, caused by the heterogeneity of concrete, lead to damage and material deterioration within the mortar matrix.

The investigations in [3] of the surface displacements shortly before failure using ARAMIS confirm a strong inhomogeneous three-dimensional stress distribution and resulting strong anisotropic damage. The results show that the internal compressive stresses might lead to a diffuse, smeared damaging effect in the mortar matrix, whereby these areas of vertical compressive displacement (in the direction of loading) are horizontally orientated and spread over the complete specimen’s width and height. From the results in [3], a strong indication of the loosening of the microstructure (detectable as a reduction of stiffness in the area of minimum stress) in these areas can be found. Shah and Chandra [9] also found loosening of the microstructure by using ultrasonic measurements. Concurrently, areas of high horizontal tensile displacement are more localised and vertically orientated.

Altogether, findings in literature [1,3,9,30] indicate that two stress-level-dependent damage mechanisms might exist: diffuse widespread compressive damage on a small scale and more localised tensile damage, visible in the late damage process on a greater scale as rather vertical-orientated cracks (in the direction of loading). Thiele [3] additionally assumes that compressive fatigue behaviour is influenced strongly by the damage effects due to high internal compressive stresses, which could not be (visibly) observed as cracks.

Overall, only a little verified knowledge concerning the damage processes in concrete subjected to compressive fatigue loading is generally available. Furthermore, new knowledge is challenging to gain due to the limits of the microstructure investigation methods. Nevertheless, hypotheses concerning damage mechanisms are available which have to be considered and checked. However, currently, the combined analyses of global macroscopic damage indicators and discrete microstructural analyses seems to be the best way for gathering further knowledge concerning damage mechanisms due to compressive fatigue loading.

In the following, the results from the experimental part of the collaborative research project “Material composition influenced damage development in high-strength concrete under cyclic loading”, with Prof. Löhnert (TU Dresden, numerical part of the project), are presented. This project is part of the DFG priority programme SPP 2020 “Cyclic Deterioration of High-Performance Concrete in an Experimental-Virtual Lab”. In this project, the influence of different high-strength concrete compositions on compressive fatigue behaviour and damage development is investigated for two maximum stress levels.

In this paper, the results of the continuously measurable macroscopic damage indicators, strain and acoustic emission hits, are presented and discussed together with the discontinuously obtainable results from microstructural analyses using light microscopy and SEM for the project’s reference high-strength concrete. One approach of the investigation of coloured resin-impregnated areas of the concrete microstructure, where damage on micro- or sub-microscale is suspected, is presented. Additionally, a clustering technique for AE-hits is applied for the first time to AE-hits that are caused by compressive fatigue loading for the purpose of characterisation. Based on the analysis of the occurrence of the types of AE-hits within the fatigue damage process, an allocation of the types of AE-hits to the damage mechanisms in the concrete microstructure is cautiously proposed. The overall objective of this paper is to provide a contribution to the knowledge concerning damage mechanisms based on a combined analysis and interpretation of the obtained results.

## 2. Materials and Methods

### 2.1. Concrete Composition and Specimen Preparation

The experimental investigations were conducted on a high-strength concrete, which is the reference concrete composition in the research project (RH1-B). The concrete composition is given in Table 1.

The water to cement (*w*/*c*) ratio is 0.35. The 28 days compressive strength tested according to DIN EN 12390-3 [33] and the modulus of elasticity tested according to DIN EN 12390-13 [34] were determined on three specimens each and the respective mean values were calculated. The mean 28 days compressive strength after storage under water is f_ck,cube,100_ = 113 MPa and the mean 28 days modulus of elasticity is E_150/300_ = 40,000 MPa. Considering the components, the mean modulus of elasticity of the coarse basalt aggregates was determined as E_basalt_ = 99,800 MPa and the modulus of elasticity of the mortar matrix as E_matrix_ = 34,900 MPa.

Cylindrical specimens with a height h = 180 mm and a diameter d = 60 mm were prepared for the fatigue tests. The specimens were cast in two layers and each layer was compacted using a vibrating table. The PVC formworks were removed 7 days after concreting and the cylinders were stored in standardised conditions (20 °C/65% R.H.) until testing. The test surfaces of the specimens were ground plane-parallel and polished to ensure a uniform stress distribution.

**Figure 2:**
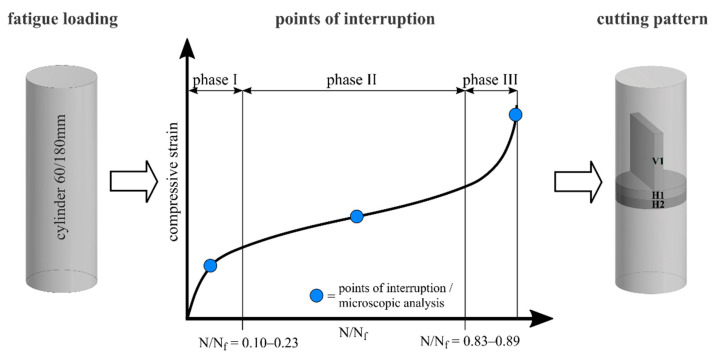
Points of interruption of partial tests and cutting pattern (schematic presentation).

All analyses of the concrete microstructure were conducted on thin sections so as to allow for transmitted light microscopy in combination with SEM. Two horizontally orientated thin sections (H1 and H2) and one vertically oriented thin section (V1) were prepared from different fatigue-loaded specimens (cf. Figure 2), thus considering a potential influence of the crack formation and internal stress state. It is known from previous investigations using the acoustic emission method, that most hits (acoustic emission hits are interpreted as damage) are located in the middle section of the specimen [13]. Hence, the thin sections were taken from the middle area of the specimens. As a reference, thin sections from two nonloaded specimens (hereinafter called phase 0) were also investigated.

To prepare the thin sections, the specimens were precut according to the cutting pattern into samples with a thickness of ≈4 mm. The samples were impregnated with blue-coloured epoxy resin (Bluedye, Struers, Willich, Germany) to enhance the visibility of cracks and pores. Afterwards, the impregnated samples were cut to format, labelled and cleaned with isopropyl and subsequently ground to around 26 µm to 28 µm thickness and then polished stepwise into thin sections. Altogether, two horizontally oriented and one vertically oriented thin sections were investigated for each fatigue phase and maximum stress level (see Figure 2 for definition). In addition, four horizontally and two vertically orientated thin sections from nonloaded specimens were under investigation.

### 2.2. Test Programme

The fatigue tests were carried out force-controlled using a class 0.5 servohydraulic testing machine with a 500 kN actuator (according to ISO 7500-1 [35]). The full amplitude was applied in the first load cycle. The minimum stress level was kept constant at S_c,min_ = 0.05 in all tests, while the maximum stress level was either S_c,max_ = 0.85 or S_c,max_ = 0.70. The test frequency applied was f_t_ = 1.0 Hz.

The compressive strength of the high-strength concrete was tested just before conducting the fatigue tests, using five specimens from the same batch having the same geometry as the specimens used in the fatigue tests. The reference compressive strength, which is required to determine the axial test forces based on the defined stress levels, was calculated as the mean value of these compressive strengths as f_cm,ref_ = 96 MPa. All fatigue tests were conducted using specimens with a minimum concrete age of 56 days.

Two different types of fatigue tests were carried out: full tests and partial tests. In the full tests, three specimens for each stress level were loaded until fatigue failure. Based on these tests, the entire fatigue behaviour until failure can be described by damage indicators. In the partial tests, fatigue loading was interrupted at predefined degradation stages to prepare samples for analyses of the microstructure (see Figure 2). The points of interruption were in the middle of phases I and II and at the end of phase III of the s-shaped fatigue strain development. The position of the transitions between the phases are given in Figure 2 as range of relative numbers of cycles to failure N/N_f_.

The middle of phase I and phase II was determined, taking into account the mean value of the phase lengths of the full tests. A constant load cycle number for test interruption in phase III cannot be applied due to the variable duration of the fatigue tests combined with the small number of load cycles in phase III. Therefore, an algorithm based on a threshold of the increase of maximum strain in phase III was developed and established in the testing machine’s control system. This allows for an automatic stop of the fatigue loading in phase III shortly before the failure of the specimen. An overview of the number of fatigue tests analysed is given in Table 2.

### 2.3. Experimental Set-Up

**Figure 3:**
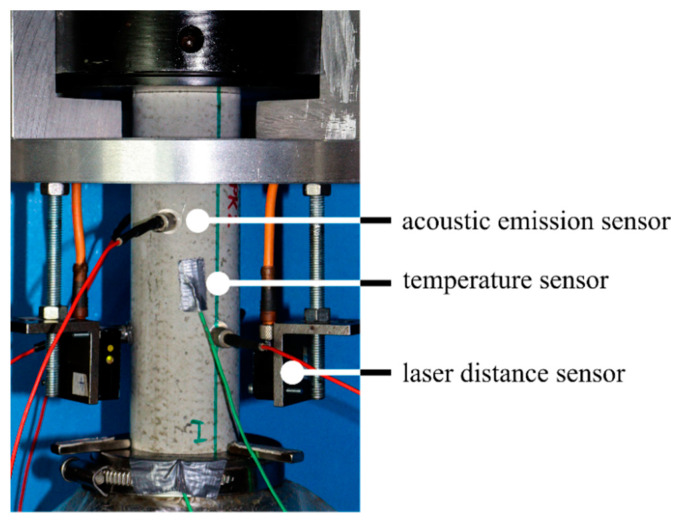
Experimental set-up.

The axial deformations in all fatigue tests were measured continuously using three laser distance sensors positioned on the circumference of the specimen at 0°, 120° and 240° (see Figure 3). In addition, the axial force, the axial displacement of the actuator, the temperature on the specimen’s surface at mid-height and the ambient temperature were measured. The sampling rate was 300 Hz for all quantities measured. Furthermore, six acoustic emission sensors were attached to the specimens. The sensors with a wideband frequency response within the range of 250 kHz to 1600 kHz were positioned at 60° from one another, alternating in the upper and lower third of the specimen. The AE-hits are characterised as single transient signals. Based on pretests, a threshold of 40 dB was defined to separate the useable signal from background noise.

### 2.4. Analysis of Macroscopic Damage Indicators

In this paper, the development of strain at maximum and minimum stress levels and the AE-hits are analysed comparatively. The three axial strains at maximum and minimum stresses were obtained by a peak analysis of the sinusoidal strain curves and afterwards averaged for each specimen. It should be mentioned that the last value of the strain developments evaluated is the peak value in the last complete load cycle and not the strain at fatigue failure. This approach was established because fatigue failure generally occurs at different stresses (but mostly in the range of the peak stress). Concurrently, a high-grade instable state of the concrete is reached in the last load cycle with strongly increasing strains. Thus, each strain at failure is connected to a different acting stress and affected by the instable state and, therefore, difficult to compare [1,2].

Results of the comparative analyses of the development of strains, stiffness (secant modulus) and dissipated energy were presented for this high-strength concrete in [13]. In this paper, the acoustic emissions are considered in addition to the strain development as another damage indicator. Here, the development of cumulated AE-hits is used to generally quantify the activity of acoustic emissions. In addition, a clustering method, considering the Gaussian mixture modelling applied to AE-hits in, e.g., [20,21] for reinforced concrete structures, is firstly applied for compressive fatigue loading. The objective is to examine the general applicability of this method, obtain additional information about the characteristics of the AE-hits occurring and obtain an indication of the type of damage developing, which is rarely visible.

**Figure 4:**
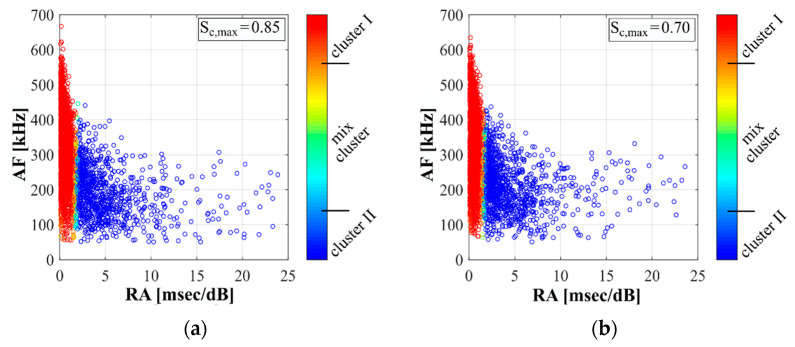
AE-hits of a full test in the RA/AF space and assignment to clusters, for S_c,max_ = 0.85 (**a**) and S_c,max_ = 0.70 (**b**).

The Gaussian mixture modelling is used to create a bivariate normal distribution based on the normal distributions of RA and AF. Thereby, each AE-hit is clearly defined as ($\vec{x}=\left(\mathrm{RA},\;\mathrm{AF}\right))$. Based on the distribution of AE-hits in the RA/AF space, the limit criterion is determined considering the posterior probability of each AE-hit to cluster I, respectively, to cluster II. It was established that the posterior probability for assignment to cluster I has to be equal or higher than 0.80. Accordingly, all hits with a posterior probability equal or lower than 0.20 are assigned to cluster II. The mix cluster contains all hits whose posterior probability is between 0.20 and 0.80. Thus, the mix cluster forms an area between both main clusters (cf. Figure 4). Based on this approach, the limit between cluster I and cluster II for the fatigue investigations conducted can be described as an almost vertical line at RA ≈ 2.0 for this application.

Figure 4 shows, as an example, the results of the clustering of AE-hits for both examined stress levels for one specimen each. It is obvious that the characteristic of the distribution of clustering results for the case of compressive fatigue loading is different compared to the schematic description in Figure 1b. It can be seen that cluster I contains AE-hits with low RA values and strongly varying AF values. The AE-hits in cluster II are characterised by higher RA values and AF values in the lower three-quarters, compared to cluster I. For analyses regarding the fatigue phases, respectively, the fatigue process up to the points of interruption, the clustering was first applied for all AE-hits occurring in the complete fatigue process N/N_f_ = 0.0–1.0 and the AE-hits were subsequently assigned to the phases, respectively, to the sections up to the points of interruption.

### 2.5. Analyses of Concrete Microstructure

Regarding the analyses of the concrete microstructure, we distinguish between the scale levels, i.e., the microscale in the range < 1 μm investigated using scanning electron microscopy SEM with secondary electron imaging, the mesoscale between 1 μm and 1000 μm investigated via light microscopy, and the macroscale >1000 μm characterised by the macroscopic damage indicators strain and AE-hits. In a first step, the thin sections were examined via digital transmitted light microscopy (VHX-7000, Keyence, Osaka City, Japan). One image with a dimension of a/b = 19.00 mm/14.26 mm and a magnification of 50× and constant illumination was acquired of each thin section. Each image represented the region of interest (ROI) located in the middle of the thin section. A total of eighteen ROIs from the preloaded microstructure were investigated, three ROIs (H1, H2 and V1) for each specimen tested in the partial tests (cf. Table 2). From two nonloaded, pristine specimens (phase 0) six ROIs were additionally investigated as references.

Although a high-strength concrete was investigated with possibly less inhomogeneous stress distribution and strengthened ITZ, and with respect to the literature, it was expected that more cracks would be visible, at least in phase III, shortly before fatigue failure. However, only a few cracks could be detected by utilising transmitted light microscopy on the mesoscale. Thus, the difficulties in detection and evaluation of cracks previously reported, e.g., in [3,9], can be confirmed. However, the ROIs showed areas of mortar matrix with accumulated blue epoxy resin with different specifications in the different phases of the fatigue process.

**Figure 5:**
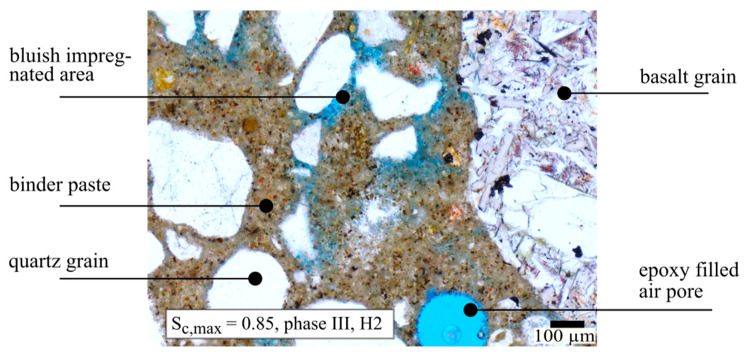
Light microscopic image of preloaded high-strength concrete (S_c,max_ = 0.85, phase III, H2). Bluish impregnated matrix areas indicate regions with a higher porosity or possible microdefects.

Figure 5 shows, as an example, a part of an ROI from a phase III thin section H2 after loading with a maximum stress level of S_c,max_ = 0.85. The brown-coloured mortar matrix is well identifiable. The multicrystalline grain on the right side of the image is identified as a basalt aggregate and the smaller grains appearing white consist mainly of quartz. The circular blue area at the bottom of the figure is the cross-section of a spherical, epoxy-filled air pore. The mortar matrix shows areas where the blue epoxy resin accumulates, well distinguishable from the homogeneous coloured air pore. These bluish impregnated areas indicate regions with a higher porosity or more microdefects. During impregnation the coloured resin fills existing pores, cracks and microstructural flaws not visible as cracks on the mesoscale. Damage on a microscale due to fatigue loading is suspected in these areas, following [1,3,30], however has not been quantified. Thus, these bluish impregnated areas were further analysed to obtain information about the distribution and development of the damage visible on the mesoscale.

Automatic segmentation of the images was not feasible because the bluish impregnated areas were not sharp-edged. Therefore, the bluish impregnated areas were marked manually using Inkscape and further automatically threshold segmented and analysed using the data visualisation software Avizo (ThermoFisher Scientific, Waltham, MA, USA). The number of segmented (i.e., bluish) areas for each ROI was counted and their respective area and total sizes were determined. The results for the horizontally orientated thin sections H1 and H2 of the same maximum stress level and fatigue phase were averaged. The vertically orientated thin sections V1 were considered separately.

Additionally, selected areas of the ROI were further evaluated using SEM (JSM 6360, Jeol, Tokyo, Japan) to obtain additional information about suspected damage in the microstructure on a microscale. Accordingly, the thin sections were carbon-coated and attached to a sample holder with conductive carbon tapes. Scanning electron microscopy images with different magnifications and constant acceleration voltage (15 kV) were taken to compare areas with and without accumulated resin. Of course, the secondary electrons give mainly topographic information of the specimen. However, there are also partly reflected electrons from the sample material due to the pear-shaped region of the interaction volume of the electron beam. Thus, additional information about the chemical composition, especially of thin sections, can be considered with caution.

## 3. Results

### 3.1. Macroscopic Damage Indicators

**Figure 6:**
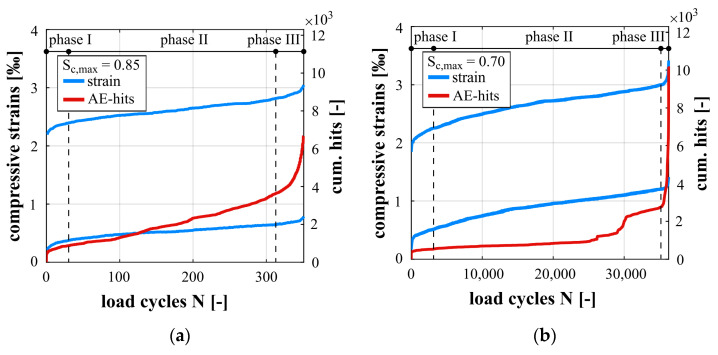
Development of maximum and minimum compressive strains and cumulated AE-hits, for S_c,max_ = 0.85 (**a**) and S_c,max_ = 0.70 (**b**).

In Figure 6, the developments of strains at maximum and minimum stress levels (blue curves) and the development of cumulated AE-hits (red curves) are presented together for S_c,max_ = 0.85 (Figure 6a) and S_c,max_ = 0.70 (Figure 6b). They are shown for one representative specimen for each stress level. In addition, the phase classification based on the strain development is marked. A quantitative analysis of the damage indicators can be found in [13,36]. The mean numbers of cycles to failure were N_f_ = 343 for S_c,max_ = 0.85 and N_f_ = 45,449 for S_c,max_ = 0.70.

The developments of strain in Figure 6 show the characteristic and well-known three-phase, s-shaped curve. The high-strength concrete has a slightly higher initial strain at a maximum stress level for S_c,max_ = 0.85 compared to the lower stress level due to the higher compressive loading. The gradient in phase II (strain increase per load cycle; mind scaling of the a-axis in Figure 6) is steeper for the higher stress level compared to the lower stress level. The quantitative evaluation of the damage indicators in [13,36] confirmed this statement. Although the gradient is steeper, the overall growth of strain within the damage process and the strain in the last cycle before failure is lower for S_c,max_ = 0.85. This can be traced back to the lower numbers of cycles to failure reached for the higher stress level, therefore, fewer strain increments were accumulated in total. Furthermore, the strains at transitions between the phases are higher for the lower stress level (cf. also [13,36]). The different strains at the transitions of phases and in the last cycles before failure reject the existence of a strain-based limit criterion and confirm results from [1,2] for another high-strength concrete.

The AE-hits occurred in phase I and II mainly in the region of the maximum stress of the sinusoidal loading and in phase III within the complete sinusoidal cycles (not shown). Shortly before failure, a significant number of AE-hits was additionally observed in the region of the minimum stress. The development of cumulated AE-hits for both stress levels shows a similar qualitative s-shape as the strain development (cf. Figure 6). First there is a short significant increase in AE-hits in phase I, followed by a continuous increase in phase II and a disproportionate increase in phase III. For S_c,max_ = 0.70, a slight stepwise increase of AE-hits can be seen in the last third of phase II. This behaviour was observed for all specimens tested on this lower stress level and, therefore, seems to be characteristic and signalises that the transition to phase III is forthcoming. Comparable progressions could not be detected neither in the strain development nor in the stiffness development (cf. [13,36]).

The gradient of cumulated AE-hits in phase II is steeper for the higher stress level, indicating greater acoustic emission activity per load cycle, and, therefore, correlates to the steeper gradient of the strain development. Despite the difference in gradient of cumulated AE-hits, a similar cumulative number of AE-hits in the range of 2900 to 3100 at the transition from phase II to phase III was found in all investigations and both stress levels for this concrete. This might be an indication of the presence of a critical accumulation of damage, which provokes the disproportional increase in strain in phase III and, thus, the fatigue failure.

**Figure 7:**
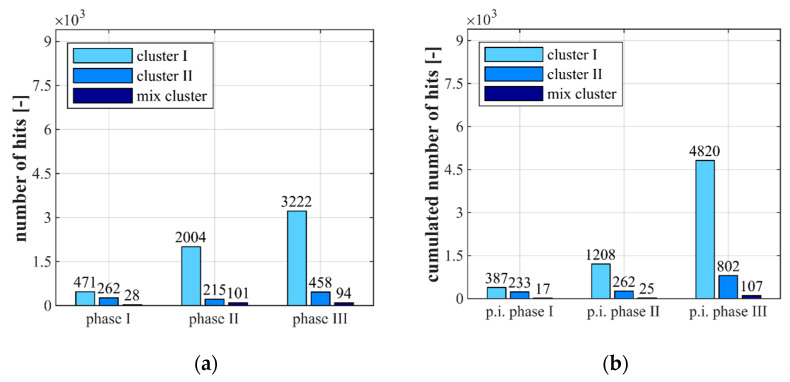
Clusters of AE-hits displayed for the entire duration of each phase (**a**) and displayed as cumulated AE-hits (i.e., including the hits from previous phases) up to the points of interruption (p.i.) (**b**) for S_c,max_ = 0.85.

**Figure 8:**
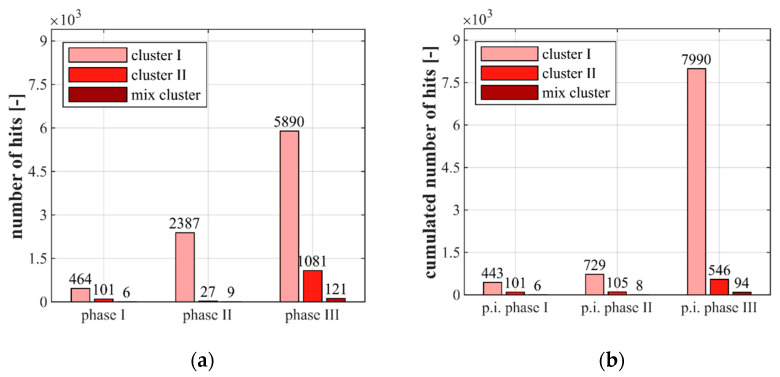
Clusters of AE-hits displayed for the entire duration of each phase (**a**) and displayed as cumulated AE-hits (i.e., including the hits from previous phases) up to the points of interruption (p.i.) (**b**) for S_c,max_ = 0.70.

Figure 7 and Figure 8 show the results of the classification of the AE-hits in clusters, as described in Section 2.3, for the stress levels S_c,max_ = 0.85 (Figure 7) and S_c,max_ = 0.70 (Figure 8). They are presented for each stress level as numbers of AE-hits within each phase (a) and as cumulated AE-hits occurring up to the points of interruption (p.i.) (b) (see Figure 2). The latter was used for the combined analyses including the microscopic analyses with respect to the manifestation of changes in the microstructure up to theses discrete points in the fatigue process. The values displayed in Figure 7 and Figure 8 were each determined as the mean values of three tests with the same stress level.

Figure 7 and Figure 8 clearly show that relatively small numbers of AE-hits are assigned to the mix cluster for both stress levels and all three phases. Furthermore, it is obvious that most AE-hits are assigned to cluster I in all phases and that a huge increase of AE-hits in cluster I takes place during the fatigue process. By contrast, AE-hits in cluster II slightly decrease in phase II compared to phase I and once again increase in phase III (Figure 7a and Figure 8a). Comparing both stress levels in Figure 7a and Figure 8a, more AE-hits in cluster II were detected in phase I and II for the higher stress level S_c,max_ = 0.85, although the absolute number of load cycles in these phases was lower compared to the lower stress level.

It is obvious from Figure 7b and Figure 8b that more AE-hits in cluster I occur up to the stops in phase I and phase II for both stress levels and that the increase of hits in both cluster I and II is more pronounced for the higher stress level. The relatively low number of cumulated AE-hits in cluster I for S_c,max_ = 0.70 up to the point of interruption in phase II (Figure 7b) compared to the total number of AE-hits in phase II (Figure 7a) can be traced back to the fact that the interruption was before the characteristic stepwise increase of hits shown previously in Figure 6b. Thus, the stepwise development of cumulated hits leads to an equalisation of the sum of hits in cluster I in phase II compared to those for S_c,max_ = 0.85.

From Figure 7a and Figure 8a it can be seen that significantly more AE-hits in cluster I were detected in phase III for S_c,max_ = 0.70. Furthermore, and interesting to note, here, also considerably more AE-hits in cluster II arose compared to the higher stress level, although considerably fewer AE-hits in cluster II occurred in the phases before. Concerning the AE-hits up to the points of interruption, significantly more AE-hits in cluster I were detected for S_c,max_ = 0.70 up to the point of interruption in phase III, but more AE-hits in cluster II were detected for S_c,max_ = 0.85 (Figure 7b and Figure 8b).

The differences in the development of cumulated AE-hits and in the cluster assignment found between the two stress levels investigated might be interpreted as different damage mechanisms occurring in the concrete microstructure due to the different stress levels. Together with the different strains at transition to phase III and the differences in the cluster assignment of the AE-hits occurring in phase I and II for both stress levels, an indication is found for the existence of a stress-level-dependent state of damage, which is reached up to the transition to phase III. However, concurrently, a similar range of a number of cumulated AE-hits (as described previously, Figure 6), which is indeed dominated by hits in cluster I, is found at the transition to phase III. Thus, a critical amount of accumulated damage might exist which initiates the transition to phase III and, therefore, the fatigue failure. However, both hypotheses do not compulsorily exclude each other.

### 3.2. Microstructural Damage Visualisation

As mentioned previously in Section 2.4, only a few cracks with widths in the range of 2 µm to 6 µm were visible by light microscopy, even for the thin sections from phase III specimens. In phase III, some cracks were found in the ITZ of the basalt aggregates, indicating detachments from the matrix. Therefore, the bluish impregnated areas in the ROIs were further evaluated and quantified (cf. Figure 5). It was generally observed that these bluish impregnated areas were especially located next to the aggregates. This might indicate that areas of high compressive stress concentration in the mortar matrix, located next to the aggregates, play a role in the fatigue damage process, as suggested by Mehmel and Kern [31].

**Figure 9:**
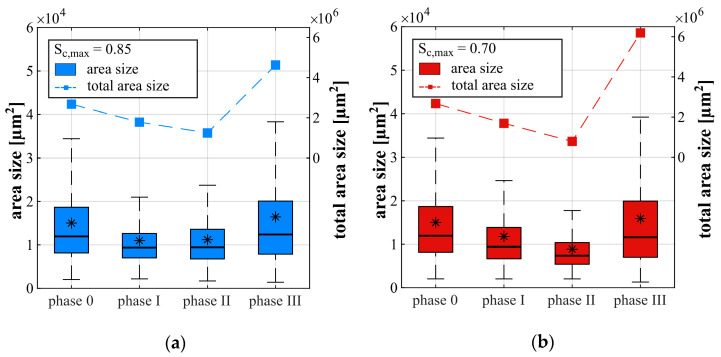
Sizes of bluish impregnated areas (boxplot, * represents the mean value) and total (cumulated) area size (dashed line) for stress level S_c,max_ = 0.85 (**a**) and S_c,max_ = 0.70 (**b**); ROIs of horizontally orientated thin sections H1 and H2.

The results of the analysis of these bluish impregnated areas or, rather, areas with suspected fatigue-induced damage on a microscale, are presented in Figure 9 for the ROIs of the horizontally orientated thin sections H1 and H2. The vertically orientated thin sections V1 were analysed separately and are not included in this figure but showed similar characteristics. The results for the maximum stress level S_c,max_ = 0.85 are shown in Figure 9a and those for the lower stress level S_c,max_ = 0.70 in Figure 9b.

Figure 9 presents the development of the total area size as dashed curves (right-hand *y*-axis) and boxplots of the single area sizes (left-hand *y*-axis) within the ROIs for each stress level. The upper and lower quantile can be seen from the coloured boxes. The median of the single area sizes is marked as a horizontal line and the mean value is marked as an asterisk. Phase 0 presents the thin sections prepared from nonloaded specimens, serving as a reference for both stress levels. The data of phases I–III displayed describes the discrete stages of the microstructure which were reached up to each point of interruption within the appropriate phase (cf. Figure 2). The results are interpreted with caution due to the limited numbers of ROIs.

It can be seen from Figure 9 that the bluish impregnated areas were already detectable for the nonloaded samples. This was expected and can be traced back to defects caused by hydration and shrinkage or, rather, sample preparation. Figure 9 shows that due to the fatigue loading, the total area size (dashed curves) decreases from a nonloaded state up to the middle of phase II and, afterwards, rapidly increases for both stress levels. In phase III, the total area sizes are higher than the original total area size in phase 0 and, therefore, are traced back to fatigue-induced damage. Furthermore, the increase of the total area size is more pronounced for the lower stress level S_c,max_ = 0.70, which correlates to the higher increase of hits in cluster I for this stress level (cf. Figure 7b and Figure 8b).

From Figure 9 it can also be seen that the single area sizes also decreases up to the middle of phase I for both stress levels. For the higher stress level S_c,max_ = 0.85, it remains almost constant up to the middle of phase II (Figure 9a). By contrast, a further decrease is observable in phase II for S_c,max_ = 0.70, although the stress level is lower (Figure 9b). An increase of single area size can be seen from phase II to phase III for both stress levels. It is interesting to note that the single area sizes in phase III are in the range of those in phase 0, but the total area size (dashed lines) is higher than in phase 0, indicating an increased number of damaged patches. Therefore, the mean number of areas is further considered (cf. Table 3).

It can be seen from Table 3 that the mean number of areas decreases up to phase II for both stress levels and strongly increases subsequently up to the interruption in phase III. Thus, the decrease in total area size up to phase II for stress level S_c,max_ = 0.70 can be traced back to both a decrease in single area size and a reduction of numbers of detectable areas. This holds true for stress level S_c,max_ = 0.85 up to phase I, whereby the total area size reduces up to phase II due to the decreasing number of areas with concurrent remaining single area sizes (mean value). From phase II to phase III, both the increase of single area size and the increase in the number of areas lead to the strong increase in total area size.

Apparently, the method applied is not able to distinguish between the bluish areas which already exist in the pristine concrete and those newly induced by the fatigue loading. It has to be assumed that both processes, consolidation or, rather, compaction and weakening, overlap, also suggested previously by Shah and Chandra [9], and lead to the overall appearance of the bluish areas evaluated here.

The differences concerning the developments of single area sizes, total area sizes and the number of areas are an additional indication of different damage processes due to the different stress levels. The reduction in single and total area size up to the middle of phase II might partly include plastic settlement in phase I and compressive consolidation/compaction, as assumed in [3,9]. It is interesting to note that despite the detection of a considerable numbers of AE-hits up to the middle of phase I and phase II (Figure 7b and Figure 8b), the total area size decreased. This could mean, on the one hand, that the decrease in area size (compaction) observed is detectable with AE-hits. On the other hand, it could mean that the fatigue damage detectable as AE-hits occurs concurrently but is only visible as (new) bluish impregnated areas (in phase III) by light microscopy on a mesoscale when a special amount of damage on a microscale or sub-microscale has been accumulated. The second hypothesis is in alignment with the hypotheses of Oneschkow [1] and Thiele [3] (cf. Section 1) and is supported by the results documented in [30].

**Figure 10:**
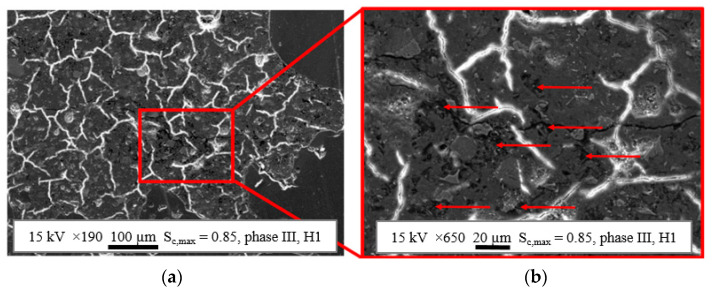
Examples of secondary electron images of concrete microstructure of the bluish impregnated area near to the aggregates at a magnification of 190× (**a**) and microstructural details at a magnification of 650× (**b**).

A further evaluation of selected areas of the ROIs was conducted using SEM, trying to identify differences between the mortar matrix of the bluish impregnated areas and the surrounding mortar matrix for each phase and stress level on a microscale. Figure 10a displays, as an example, a secondary electron image of a part of an ROI from a thin section of phase III, preloaded on the higher stress level S_c,max_ = 0.85, at a magnification of 190×. Figure 10b displays a zoom at a magnification of 650×, which was bluish impregnated under the light microscope at mesoscale.

The white appearing cracks passing through the cement matrix without alignment to each other or to the direction of fatigue loading are interpreted as shrinkage cracks, which arose after carbon coating due to the dehydration of the cement matrix under the high vacuum of the SEM [37]. The homogeneous grey particles without shrinkage cracks in Figure 10a on the right and lower edge represent sand aggregates. The horizontal crack running through Figure 10b and further on develops in Figure 10a cannot be unequivocally allocated to fatigue loading. Possible other causes could be the sample preparation or shrinkage. The evaluation generally showed that only a few cracks could be seen even in phase III, even by SEM, shortly before fatigue failure.

The structural details of the bluish impregnated areas in phase III become more visible in Figure 10b. The dark grey to black spots appearing (marked with arrows) can be assigned to the cross-sections of voids, partly or totally filled with epoxy resin. Areas where these voids accumulate correlate with the bluish impregnated areas visible by light microscopy. However, further analyses of the SEM images showed that epoxy resin also accumulated outside the bluish areas throughout the mortar matrix of each specimen in a reduced concentration. This could be identified in each specimen and every phase (phase 0 to phase III). Thus, microstructural voids also appeared in nonloaded samples of phase 0, whereby they were mostly located near to the aggregates. In samples of phase 0, a major part of these voids could be assigned to the remaining vacant capillary pores, which were filled up during epoxy impregnation.

Although only tiny evaluation areas could be analysed at microscale due to the higher magnification of the SEM compared to light microscopy, alterations of the microstructure within the fatigue process, also outside the bluish areas, were observed. A considerable increase of voids in the mortar matrix next to but also distant from the aggregates were found, especially for phase III specimens. Of course, the cracks in the ITZ of the basalt aggregates, mentioned previously, were also observable by SEM. Altogether, indication exists that the complete mortar matrix suffers material degradation in the course of fatigue loading, but is more concentrated in the bluish impregnated areas located mostly next to the aggregates. In all probability, the existence of microstructural inhomogeneities and defects in the pristine concrete microstructure influences the ongoing fatigue degradation. It is conceivable that the degradation is sufficient for the appearance of damaged areas on a mesoscale scale only where high stresses occur, due to the heterogeneity of the microstructure.

## 4. Discussion

In this section, the results of the macroscopic damage indicators, strain and acoustic emissions, and the results of the microstructural analyses by light microscopy and SEM are superordinately considered and discussed together.

As described in [31,32] for normal strength concretes, the inhomogeneous stress distribution due to the heterogeneity of the concrete microstructure, influenced, inter alia, by the stiffness differences of coarse aggregates and matrix, leads to areas with stresses much higher than the external subjected stresses, especially for high stress levels. Although a high-strength concrete is investigated in this paper with a possibly less pronounced heterogeneity due to the higher modulus of elasticity and lower porosity of the mortar matrix, the smaller ratio in volume of coarse aggregates to mortar matrix and the smaller maximum grain size of coarse aggregates, inhomogeneous stress distribution is still present.

The increase of bluish impregnated areas in phase III can be reliably traced back to fatigue-induced damage, based on a comparison with the previous phases 0–II. It was observed that these bluish impregnated areas are especially located next to the aggregates. This might indicate that areas of high compressive stress concentration in the mortar matrix, located next to the aggregates, indeed play a role in the fatigue damage, as assumed by [31]. However, it has to be assumed that these bluish areas only show the tip of the iceberg, meaning that enough damage on a microscale or lower has been accumulated in those areas so that it is visible as coloured mortar matrix on a mesoscale.

The continuously occurring great amount of AE-hits, especially of AE-hits in cluster I in phase I and phase II, together with the accruement of microstructural defects in the form of voids inside and outside the bluish areas in the mortar matrix found by SEM, indicate that the complete mortar matrix suffers fatigue-induced damage within the fatigue process. Based on this, it can be assumed that diffuse sub-micrometre damage is widespread in the mortar matrix (but more accumulated in highly stressed areas). Schaan et al. observed such nanoscale damage by using TEM [30]. However, it must be noted that it is not clear whether every accruement of damage, especially on a very low scale, is detectable with AE.

The differences in the development of cumulated AE-hits, especially in phase II, (cf. Figure 6), the differences in the development of bluish area sizes (cf. Figure 9) and the differences in the cluster assignment in all phases (cf. Figure 7 and Figure 8), together give a strong indication for the existence of two stress-level-dependent damage processes or damage mechanisms and, thus, fit the assumptions of [9]. Due to the stronger pronouncement of AE-hits in cluster II in phase I and II for S_c,max_ = 0.85 compared to the lower stress level, a different stress-level-dependent damage state at the transition to phase III is conceivable in addition. However, concurrently, a similar sum of AE-hits, dominated by AE-hits in cluster I, was detected for both stress levels up to the transition to phase III. This is an indication of the existence of a critical amount of damage, which initiates the transition and, therefore, the fatigue failure. However, both hypotheses do not compulsorily exclude each other.

Findings in the literature [1,3,9,30] indicate the existence of two damage mechanisms: diffuse widespread compressive damage on a small scale and more localised tensile damage, visible in the late damage process on a greater scale as rather vertical-orientated cracks (in the direction of loading). Considering the findings from literature together with the results of AE-clustering—dominance of hits in cluster I, a more pronounced occurrence of hits in cluster II for the higher stress level and mainly in phase III—and together with the SEM results of the spread accruement of fatigue-induced voids in the mortar matrix on a microscale, it is conceivable that hits in cluster I might result from spread diffuse compressive sub-microscale damage. Cluster II might include hits caused by localised tensile damage on the microscale and greater, especially in phase III. It has to be clearly stated that this is a very first attempt to find an allocation of the characteristics of AE-hits to different forms of compressive fatigue damage, which definitely has to be checked in further investigations.

## 5. Summary and Conclusions

Investigations on the fatigue behaviour and the damage processes in a high-strength concrete are presented in this paper. Fatigue tests with two maximum stress levels of S_c,max_ = 0.85 and S_c,max_ = 0.70 with a constant minimum stress level of S_c,min_ = 0.05 and a frequency of f_t_ = 1.0 Hz were conducted up to fatigue failure and up to defined points of interruption in the fatigue process. Thin sections were prepared for the microstructural analyses and analysed using transmitted light microscopy and SEM. In addition, the macroscopic damage indicators, strain and acoustic emission, were considered in order to obtain more global information about the damage process and combine them with information only obtainable locally about the damage in the microstructure.

Higher strain increase and more AE-hits per load cycle in phase II were found for the higher stress level, whereas the total increase in strain and the total sum of hits within the fatigue process were lower compared to the lower stress level due to the lower number of cycles to failure. A stepwise increase of AE-hits in the last third of phase II was observed for the lower stress level (S_c,max_ = 0.70), which seems to be characteristic and signalises the transition to phase III to be forthcoming. However, despite differences in AE-hits per load cycle, a similar range of cumulated AE-hits at the transition to phase III was found for both stress levels, which might indicate the existence of a certain threshold for a critical accumulation of damage. The classification of AE-hits based on a Gaussian mixture modelling in two clusters was applied for the first time to compressive fatigue loading of concrete. The results showed that the AE-hits occurring were predominately located in cluster I over the complete fatigue process. The AE-hits in cluster II appeared especially for the higher stress level and in phase III for both stress levels.

Only a few cracks were visible using transmitted light microscopy and SEM, even in phase III shortly before fatigue failure. However, areas in the mortar matrix with accumulated bluish epoxy resin due to higher porosity or microstructural defects were further investigated. Their total area size and numbers decreased, stress level dependently, up to the middle of phase II and then increased strongly up to a higher number than was found in the reference samples from nonloaded specimens. The development of the bluish area is currently associated with changes in porosity in the sub-micron or sub 10 µm range (note: at this scale level, no distinction can be made between pores and cracks).

It was observed by SEM on the microscale that voids were located in these bluish areas. Furthermore, an increase in the number of microstructural voids was found especially in phase III inside and outside the bluish areas, indicating that the complete mortar matrix suffers material degradation and that the bluish areas, visible by microscopy, are only the tip of the iceberg with a greater amount of accumulated damage.

Considering the findings and assumptions documented in literature [1,3,9,30,31] together with the results of the investigations presented in this paper, indications for the following hypotheses were found:

- The mortar matrix might suffers diffuse and widespread sub-microscale compressive damage within the complete compressive fatigue process, whereby more damage might be accumulated in highly stressed areas. In addition, localised tensile damage might develop, especially visible in the late fatigue process as cracks at microscale and greater.
- Widespread compressive damage might dominate the fatigue process, especially in the first two phases, whereby the dominance against localised tensile damage might increase with a decreasing maximum stress level (stress-level dependency).
- A stress-level-dependent state of damage might be reached at the transition from phase II to phase III of the strain development, whereby a critical amount of induced damage might concurrently lead to this transition.
- In this investigation, the hits in cluster I might be assigned to diffuse and widespread sub-microscale compressive damage. Concurrently, cluster II might include AE-hits caused by localised tensile damage on the microscale and greater.

It should be mentioned that, of course, the pronouncement of damage mechanisms is additionally dependent on the heterogeneity of the concrete microstructure and the inhomogeneity of stress distribution influenced hereby. The hypotheses stated previously have to be examined and proved in further investigations and discussed in the research community. However, the results presented in this paper clearly show that the combined analyses of macroscopic damage indicators (here, strain and AE-hits) together with microstructural analyses can lead to new knowledge concerning the occurrence and development of fatigue damage in the concrete microstructure. Especially the classification or, rather, clustering of AE-hits should be further improved for the case of compressive fatigue loading of (small-scaled) concrete specimens. This is currently further under investigation. Overall, the results presented also demonstrate that enormous effort is necessary to obtain new knowledge due to the currently still existing limits of micro- and nanostructural investigation techniques, resulting in putting together the findings like small puzzle pieces.

## Figures and Tables

**Table 1:** Composition of the investigated concrete RH1-B.

Component	Quantity
Portland Cement (CEM I 52, 5 R HS/NA)	446.60 kg/m^3^
Quartz sand (0/0.5 mm)	75.00 kg/m^3^
Sand (0/2 mm)	850.00 kg/m^3^
Basalt (2/5 mm)	350.00 kg/m^3^
Basalt (5/8 mm)	570.00 kg/m^3^
PCE plasticiser	5.00 kg/m^3^
Stabilizer	2.85 kg/m^3^
Water	176.00 kg/m^3^

**Table 2:** Number of fatigue tests analysed (full and partial tests).

Test Procedure	Number of Fatigue Tests [-]	
	S_c,max_ = 0.85	S_c,max_ = 0.70
Full tests	3	3
Partial tests		
Phase I	1	1
Phase II	1	1
Phase III	1	1

**Table 3:** Mean numbers of bluish impregnated areas at points of interruption.

Phases	Number of Bluish Impregnated Areas [-]	
	S_c,max_ = 0.85	S_c,max_ = 0.70
Phase 0	808	808
Phase I	686	605
Phase II	471	330
Phase III	1170	1513

## Data Availability

Data available on request.
